# Triple combination antibiotic therapy for carbapenemase-producing *Klebsiella pneumoniae*: a systematic review

**DOI:** 10.1186/s12941-017-0249-2

**Published:** 2017-11-25

**Authors:** David M. Jacobs, M. Courtney Safir, Dennis Huang, Faisal Minhaj, Adam Parker, Gauri G. Rao

**Affiliations:** 1 Department of Pharmacy Practice, University at Buffalo School of Pharmacy and Pharmaceutical Sciences, Buffalo, NY USA; 2 Division of Pharmaceutics and Experimental Therapeutics, Eshelman School of Pharmacy, University of North Carolina, Chapel Hill, NC USA

**Keywords:** Triple combination treatment, KPC, Antibiotic resistance, Carbapenemase-producing *K. pneumoniae*

## Abstract

**Background:**

The spread of carbapenemase-producing *K. pneumoniae* (CPKP) has become a significant problem worldwide. Combination therapy for CPKP is encouraging, but polymyxin resistance to many antibiotics is hampering effective treatment. Combination therapy with three or more antibiotics is being increasingly reported, therefore we performed a systematic review of triple combination cases in an effort to evaluate their clinical effectiveness for CPKP infections.

**Methods:**

The PubMed database was searched to identify all published clinical outcomes of CPKP infections treated with triple combination therapy. Articles were stratified into two tiers depending on the level of clinical detail provided. A tier 1 study included: antibiotic regimen, regimen-specific outcome, patient status at onset of infection, and source of infection. Articles not reaching these criteria were considered tier 2.

**Results:**

Thirty-three studies were eligible, 23 tier 1 and ten tier 2. Among tier 1 studies, 53 cases were included in this analysis. The most common infection was pneumonia (31%) followed by primary or catheter-related bacteremia (21%) and urinary tract infection (17%). Different combinations of antibiotic classes were utilized in triple combinations, the most common being a polymyxin (colistin or polymyxin B, 86.8%), tigecycline (73.6%), aminoglycoside (43.4%), or carbapenem (43.4%). Clinical and microbiological failure occurred in 14/39 patients (35.9%) and 22/42 patients (52.4%), respectively. Overall mortality for patients treated with triple combination therapy was 35.8% (19/53 patients).

**Conclusions:**

Triple combination therapy is being considered as a treatment option for CPKP. Polymyxin-based therapy is the backbone antibiotic in these regimens, but its effectiveness needs establishing in prospective clinical trials.

## Background

The increasing global prevalence of carbapenem-resistant Enterobacteriaceae (CRE) combined with the decline in effective therapies is a public healthcare crisis. Infections caused by these multi-drug resistant (MDR) Gram-negative bacteria are associated with high mortality rates, often estimated at 40% or higher [1–7]. Since its discovery, CRE has spread to every continent and is now endemic in certain areas [1, 8–10]. A common mechanism of resistance in CRE is the production of carbapenemase enzymes, which confer resistance to many of the currently available antibiotics and limit treatment options [3, 8, 11, 12]. Among Enterobacteriaceae, carbapenemase production is common in *Klebsiella pneumoniae* [3, 8, 9]. The concurrent administration of multiple antibiotic agents can increase pharmacodynamic killing activity and potentially suppress or delay the emergence of resistance by broadening the spectrum of activity and exploiting different mechanisms of action [2, 4, 11, 13]. In the absence of evidence-based treatment guidelines, clinicians are increasingly resorting to using combination therapy for difficult-to-treat infections on the basis of some weak but promising published data [2, 4, 6, 11].

There is currently no consensus on the most appropriate treatment for infections caused by carbapenemase-producing *K. pneumoniae* (CPKP) [3, 6]. A recent review showed a mortality benefit with the use of combination therapy for CPKP, which is encouraging [11]. However, the evidence is conflicting, as a different comprehensive review found similar mortality in patients whether treated with monotherapy or combination therapy [13]. As the minimum inhibitory concentrations (MICs) to many antibiotics continue to creep upwards and with recent reports of polymyxin resistance, treating infections caused by carbapenemase producers is increasingly challenging [14, 15] and may necessitate combination therapy of three or more antibiotics in the near future. In vitro studies have shown promising results for the treatment of highly resistant KPC-producing organisms utilizing triple drug combinations [16, 17], but clinical studies on the treatment of CPKP infections utilizing three or more antibiotics are scarce and mainly limited to case reports or small numbers of cases within larger studies. Therefore, we performed a systematic review of individual cases in an effort to evaluate the effectiveness of combination treatment regimens of three or more antibiotics (triple combination) on clinical outcomes for CPKP infections.

## Methods

### Literature search

A systematic review was conducted using the PubMed database from inception to March [1], 2016 using the following search terms: “carbapenemase-producing *Klebsiella pneumoniae*”, “carbapenem-resistant *Klebsiella pneumoniae*”, and “KPC”. Only articles published in English or translated to English were evaluated. The bibliographies of all eligible studies were reviewed in an effort to identify other eligible studies.

### Study selection

Articles were eligible if they included any patients with infections due to carbapenemase-producing *K. pneumoniae* treated with triple combination antimicrobial therapy for a minimum of 48 h. When an article included patients treated with triple combination therapy along with mono or dual antimicrobial therapy, only the triple combination data were extracted. Articles were excluded from further review if they fulfilled any of the following criteria: (1) the study evaluated *Enterobacteriaceae* other than *K. pneumoniae*; (2) the study included data based on only in vitro or in vivo infection models; (3) the details regarding the treatment regimens were not specified or included in the article; and (4) if the study only reported on patients colonized with CPKP.

Following initial identification, all potential articles were reviewed to assess their eligibility based on treatment and microbiology-specific exclusion criteria. Articles were ineligible if they fulfilled any of the following criteria: (1) carbapenemase production was not confirmed; (2) data related to CPKP could not be separated from other carbapenemase-producing Enterobacteriaceae; and (3) details regarding treatment with monotherapy or dual combination therapy specifically for the CPKP infection were included. One article was excluded as an updated version of the same study population was published soon afterwards. The authors were contacted in cases where details regarding the concurrent administration of antibiotics as a part of the triple combination were not clearly stated or could not be verified.

### Data extraction and definitions

The data extracted from each article included the main characteristics of the: (1) study (first author name, publication year, country of origin, study period and design); (2) case (including age, sex, type of infection, and APACHE II score); and (3) antibiotic treatment. Antimicrobial agents were categorized based on antibiotic class: polymyxins, carbapenems, tigecycline, aminoglycosides, beta-lactam plus beta-lactamase inhibitors, fosfomycin, trimethoprim–sulfamethoxazole, and fluoroquinolones. Clinical outcomes including clinical failure, microbiological failure and mortality were also recorded. Clinical and microbiological failure were defined according to the definitions used by the investigators of the included study. Any indeterminate outcome as listed by the study authors was categorized as a failure. Overall mortality as reported by the study authors was also recorded.

## Results

**Fig. 1:**
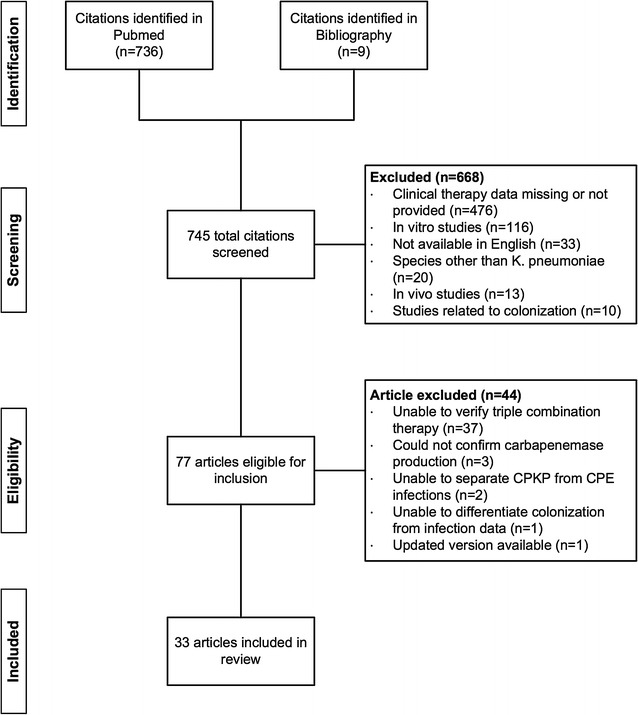
Flow diagram of the search strategy and articles selected for review. CPKP, carbapenemase-producing *K. pneumoniae*; CPE, carbapenemase-producing Enterobacteriaceae

The results of the literature search and the process of selection of included publications are shown in Fig. 1. A total of 736 articles were retrieved from PubMed using the search terms listed, and 668 articles were excluded because articles were not available in English (n = 33), Enterobacteriaceae other than *Klebsiella pneumoniae* were studied (n = 20), in vitro (n = 116) and in vivo (n = 13) models of infection were used, details regarding the clinical therapy were missing or not provided (n = 476), and publications were not related to patients colonized with bacteria of interest (n = 10). Based on the initial search criteria, nine articles were retrieved based on our review of the bibliographies of articles included. Based on the second set of exclusion criteria, 44 additional publications were excluded because we could not: (1) confirm carbapenemase production (n = 3), (2) separate CPKP data from other CRE infections (n = 2), (3) separate colonization data from infection data (n = 1), and (4) verify concurrent administration of three agents (n = 37).

**Fig. 2:**
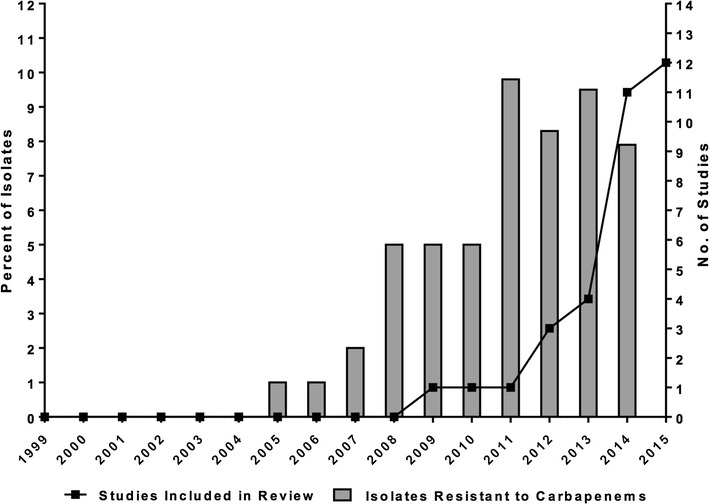
Trends in the number of studies included in this review by publication year and the percentage of carbapenem-resistant *K. pneumoniae* isolates in the United States. Percent of resistant isolates is a composite of data from the Center for Disease Dynamics, Economics & Policy and the Centers for Disease Control and Prevention

**Fig. 3:**
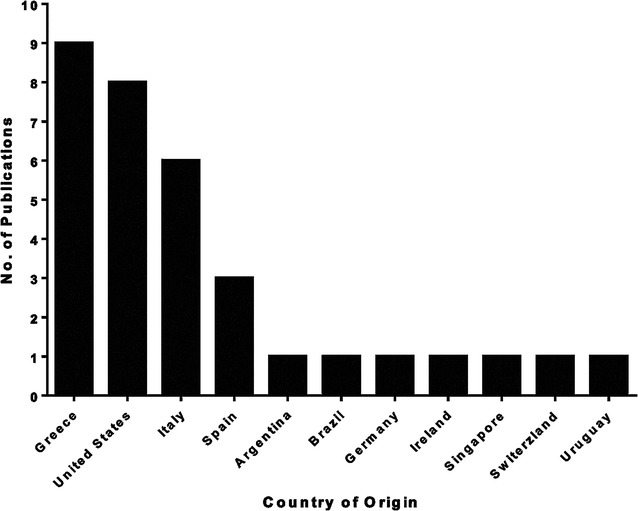
Studies included in the review by country of origin (n = 33)

The remaining 33 studies satisfied the minimum treatment duration requirement and were included in our final analysis. Thirteen studies were a combination of case reports or case series [18], fifteen were cohort studies [12, 31], and five were case–control studies [15, 45]. Study publication years ranged from 2009–2015, with the number of publications increasing in frequency over time, which coincided with an increasing trend in the percent of carbapenem-resistant *K. pneumoniae* isolates in the United States (Fig. 2). The country of origin for most of these studies was Greece (n = 9), the United States (n = 8), and Italy (n = 6) (Fig. 3).

The articles included in this review were stratified into two tiers depending on the details of the reported treatment. A study classified as tier 1 included all of the following information: (1) antibiotic regimen, (2) regimen-specific outcome, (3) regimen-specific patient status at onset of infection, and (4) source of the infection. A study classified as tier 2 lacked information regarding one or more of these critical elements.

### Tier 1 results

#### Patient characteristics

Twenty-three studies were classified as tier 1 [18–33, 37–41, 43, 47] and comprised 53 patients (Table 1). The characteristics of these patients are presented in Table 2. The mean age was 54 ± 16 years and 67% were males. Seventeen patients (63%) had an APACHE II score ≥ 15 and the median SOFA score was 10 (range 3–15). At time of onset of infection, six (25%) patients were septic and 12 (50%) patients had severe sepsis or septic shock.

**Table 1:** Summary of outcomes and antimicrobial therapy for carbapenemase-producing *K. pneumoniae* infections found in tier 1 articles

First Author (no. of subjects) [References]	Clinical failure^d^	Microbiologic failure^d^	Mortality	Combination antimicrobial therapy						
				PMB/COL	TCN	AMG	CARB	FOS	PCN	FQ
Ariza-Heredia (n = 1) [18]	–	1/1 (100%)	0/1 (0%)	0/1 (0%)	1/1 (100%)	1/1 (100%)	0/1 (0%)	0/1 (0%)	0/1 (0%)	1/1 (100%)
Balandin Moreno (n = 3) [31]	–	2/3 (66.7%)	0/3 (0%)	2/3 (66.7%)	3/3 (100%)	1/3 (33.3%)	2/3 (66.7%)	0/3 (0%)	0/3 (0%)	1/3 (33.3%)
Camargo (n = 1) [19]	–	1/1 (100%)	0/1 (0%)	1/1 (100%)	1/1 (100%)	0/1 (0%)	1/1 (100%)	0/1 (0%)	0/1 (0%)	0/1 (0%)
Chua^a^ (n = 2) [20]	0/2 (0%)	0/2 (0%)	0/2 (0%)	2/2 (100%)	0/2 (0%)	0/2 (0%)	2/2 (100%)	0/2 (0%)	0/2 (0%)	0/2 (0%)
Cicora (n = 5) [32]	–	4/5 (80%)	1/5 (20%)	3/5 (60%)	5/5 (100%)	0/5 (0%)	3/5 (60%)	4/5 (80%)	0/5 (0%)	0/5 (0%)
Clancy (n = 1) [33]	0/1 (0%)	0/1 (0%)	0/1 (0%)	1/1 (100%)	1/1 (100%)	1/1 (100%)	1/1 (100%)	0/1 (0%)	0/1 (0%)	0/1 (0%)
de Sanctis (n = 1) [21]	–	1/1 (100%)	1/1 (100%)	1/1 (100%)	1/1 (100%)	1/1 (100%)	0/1 (0%)	0/1 (0%)	0/1 (0%)	0/1 (0%)
Giamarellou (n = 1) [22]	1/1 (100%)	1/1 (100%)	0/1 (0%)	1/1 (100%)	0/1 (0%)	1/1 (100%)	1/1 (100%)	1/1 (100%)	0/1 (0%)	0/1 (0%)
Lemmenmeier (n = 3) [23]	0/3 (0%)	–	0/3 (0%)	3/3 (100%)	3/3 (100%)	0/3 (0%)	3/3 (100%)	0/3 (0%)	0/3 (0%)	0/3 (0%)
Lubbert (n = 4) [37]	3/4 (75.0%)	–	3/4 (75%)	4/4 (100%)	4/4 (100%)	4/4 (100%)	0/4 (0%)	0/4 (0%)	0/4 (0%)	0/4 (0%)
Maltezou (n = 2) [38]	0/2 (0%)	2/2 (100%)	0/2 (0%)	2/2 (100%)	2/2 (100%)	2/2 (100%)	0/2 (0%)	0/2 (0%)	0/2 (0%)	0/2 (0%)
Marquez^b^ (n = 1) [24]	1/1 (100%)	1/1 (100%)	1/1 (100%)	0/1 (0%)	1/1 (100%)	0/1 (0%)	1/1 (100%)	0/1 (0%)	0/1 (0%)	0/1 (0%)
Mathers^c^ (n = 1) [25]	0/1 (0%)	0/1 (0%)	0/1 (0%)	1/1 (100%)	0/1 (0%)	1/1 (100%)	0/1 (0%)	0/1 (0%)	1/1 (100%)	0/1 (0%)
Morris (n = 1) [39]	0/1 (0%)	–	0/1 (0%)	1/1 (100%)	1/1 (100%)	1/1 (100%)	0/1 (0%)	0/1 (0%)	0/1 (0%)	0/1 (0%)
Mouloudi (n = 4) [40]	1/4 (25.0%)	1/4 (25%)	2/4 (50%)	4/4 (100%)	4/4 (100%)	4/4 (100%)	0/4 (0%)	0/4 (0%)	0/4 (0%)	0/4 (0%)
Navarro (n = 2) [41]	–	–	2/2 (100%)	2/2 (100%)	2/2 (100%)	2/2 (100%)	0/2 (0%)	0/2 (0%)	0/2 (0%)	0/2 (0%)
Nevrekar (n = 1) [26]	0/1 (0%)	0/1 (0%)	0/1 (0%)	1/1 (100%)	1/1 (100%)	1/1 (100%)	0/1 (0%)	0/1 (0%)	0/1 (0%)	0/1 (0%)
Oliva^a^ (n = 1) [27]	0/1 (0%)	0/1 (0%)	0/1 (0%)	1/1 (100%)	0/1 (0%)	0/1 (0%)	1/1 (100%)	0/1 (0%)	0/1 (0%)	0/1 (0%)
Pontikis (n = 9) [28]	5/9 (55.6%)	4/9 (44.4%)	4/9 (44.4%)	8/9 (88.9%)	4/9 (44.4%)	2/9 (22.2%)	3/9 (33.3%)	9/9 (100%)	2/9 (22.2%)	0/9 (0%)
Sanchez-Romero (n = 1) [47]	–	–	0/1 (0%)	1/1 (100%)	1/1 (100%)	1/1 (100%)	0/1 (0%)	0/1 (0%)	0/1 (0%)	0/1 (0%)
Souli (n = 6) [43]	3/6 (50%)	3/6 (50%)	5/6 (83.3%)	6/6 (100%)	3/6 (50%)	3/6 (50%)	4/6 (66.7%)	0/6 (0%)	2/6 (33.3%)	2/6 (33.3%)
Viaggi (n = 1) [29]	0/1 (0%)	1/1 (100%)	0/1 (0%)	1/1 (100%)	1/1 (100%)	0/1 (0%)	0/1 (0%)	1/1 (100%)	0/1 (0%)	0/1 (0%)
Virgilio (n = 1) [30]	0/1 (0%)	0/1 (0%)	0/1 (0%)	1/1 (100%)	0/1 (0%)	0/1 (0%)	1/1 (100%)	1/1 (100%)	0/1 (0%)	0/1 (0%)
N = 53	14/39 (35.9%)	22/42 (52.4%)	19/53 (35.8%)	47/53 (88.6%)	39/53 (73.6%)	26/53 (49.1%)	23/53 (43.4%)	16/53 (30.2%)	5/53 (9.4%)	4/53 (7.5%)

*PMB* polymyxin B, *COL* colistin, *TCN* tetracycline, *AMG* aminoglycoside, *CARB* carbapenem, *FOS* fosfomycin, *PCN* penicillin, *FQ* fluoroquinolone

^a^Dual carbapenem therapy used

^b^Rifampin also administered

^c^Trimethoprim–sulfamethoxazole also administered

^d^Dash indicates information was unavailable

**Table 2:** Patient characteristics among tier 1 articles (n = 53)

Characteristic	
Age (n = 53)^a^	54 ± 16^b^
Male Sex, n (%) [n = 46]^a^	31 (67)
ICU admission, n (%) [n = 38]	33 (87)
APACHE II Score ≥ 15, n (%) [n = 27]^a^	17 (63)
SOFA (n = 18)^a^	10 (3–15)^c^
Sepsis syndrome at onset of infection, n (%) (n = 24)	
Sepsis	6 (25)
Severe sepsis or septic shock	12 (50)

*APACHE II* Acute Physiology and Chronic Health Evaluation II, *SOFA* Sequential Organ Failure Assessment

^a^Number of study subjects for which this data were available

^b^Presented as mean ± standard deviation

^c^Presented as median (range)

#### Clinical outcomes and antibiotic utilization

Outcomes and antibiotic utilization stratified by publication for all patients in tier 1 are reported in Table 1. Of the 53 patients included in tier 1, clinical failure data were available for 39 patients, and 14 of these (35.9%) failed on triple combination therapy. Microbiologic failure data were available for 43 patients, and 22 of these (52.4%) failed on triple combination therapy. Overall, crude mortality among these patients was 35.8% (19/53). Polymyxins were the most frequently (47/53 patients, 88.6%) administered class of antibiotics among the different combination therapies followed by tigecycline (39/53 patients, 73.6%), aminoglycosides (26/53 patients, 49.1%), and carbapenems (23/43 patients, 43.4%). Rifampin and trimethoprim–sulfamethoxazole were used in one case each (1.9%), with dual carbapenem therapy being utilized in three patients (5.7%). Triple combination therapy was initiated empirically in six patients (11.3%) and was provided as definitive treatment in 21 patients (39.6%). Treatment sequence was not provided in 26 patients (49%).

#### Antimicrobial regimens by infection source

Patient-specific antimicrobial regimens and outcomes stratified by infection source are presented in Table 3. Pneumonia was the most commonly reported (31%) infection followed by primary or catheter-related bacteremia (21%) and urinary tract infections (17%). For pneumonia, patients were most commonly treated with a polymyxin (16/17 patients, 94%) followed by tigecycline (13/17 patients, 76%). Overall, four patients with pneumonia died (24%) either on or following treatment with combination antibiotic therapy. For the treatment of primary or catheter-related bacteremia, nine patients (9/11 patients, 82%) were treated with a polymyxin followed by meropenem (7/11 patients, 64%). The crude mortality was 55% (6/11 patients) for patients with bacteremia. For the treatment of a urinary tract infection, polymyxin was commonly utilized (7/9 patients, 78%) followed by meropenem (5/9 patients, 56%). Two patients (22%) treated with combination therapy for a urinary tract infection died.

**Table 3:** Patient-specific summary of demographics, clinical outcomes, and combination treatment by infection source among tier 1 articles

Infection	Patient no.	Age	*bla*	Co-infection	Treatment	Microbiological outcome	Mortality
Pneumonia	1	54	KPC	–	MER COL TIG	–	No
	2	67	VIM-1	–	MER COL TIG	–	No
	3	39	VIM-1	Bacteremia	MER COL TIG	–	No
	4	22	VIM-1	–	MER COL TIG	Failure	No
	5	52	KPC	–	GEN COL TIG	–	No
	6		KPC	–	GEN COL TIG	–	No
	7	68	KPC	–	GEN COL TIG	Failure	No
	8	19	KPC	–	GEN COL TIG	Failure	No
	9	57	KPC-2	UTI	GEN COL TIG	–	Yes
	10	63	KPC-2	–	GEN COL TIG	–	Yes
	11	52	KPC-2	–	FOS COL TIG	Success	No
	12	28	KPC-2	–	FOS COL TIG	Failure	No
	13	77	KPC-2	Bacteremia, SSI	ERT DOR PMB	Success	No
	14	62	KPC-2	–	ERT DOR COL PMB	Success	No
	15	62	KPC-2	Bacteremia	DOR GEN COL TIG	Success	No
	16	67	KPC-2	–	MER TIG RIF	Failure	Yes
	17	72	KPC-2	–	MER FOS COL	Failure	Yes
Urinary tract	18	75	OXA-48	Bacteremia	MER ERT COL	Success	No
	19	63	KPC-2	–	MER FOS COL	Failure	No
	20	68	–	–	MER FOS COL DOX	Failure	No
	21	33	–	–	MER FOS DOX	Success	No
	22	31	–	Bacteremia	MER COL TIG	Failure	No
	23	62	–	Bacteremia	FOS COL TIG	Failure	No
	24	54	KPC	Bacteremia	DOR GEN FOS COL	Failure	No
	25	61	KPC	Bacteremia	FOS TIG DOX	Failure	Yes
	26	70	KPC-2	Bacteremia	AMK COL TIG	–	Yes
Primary or catheter-related bacteremia	27	61	KPC-2	–	MER TIG CIP	Failure	No
	28	30	KPC-2	–	MER FOS COL	Success	No
	29	42	KPC-2	–	MER GEN COL	Success	Yes
	30	69	KPC-2	–	MER COL CIP	Success	No
	31	73	KPC-2	–	GEN FOS TIG	Success	No
	32	46	KPC-2	–	AMK COL TIG TZP	Success	Yes
	33	64	KPC-2	–	MER COL TIG	Failure	No
	34	55	KPC-2	–	MER COL TIG	Failure	Yes
	35	74	VIM-1	–	MER COL TIG	Failure	Yes
	36	63	KPC-2	–	FOS COL TIG	Failure	Yes
	37	65	KPC-2	–	COL CIP TZP	Failure	Yes
Intra-abdominal	38	44	KPC-2	Bacteremia	GEN COL TIG	Success	No
	39	60	KPC-2	Bacteremia, SSI	GEN COL TIG	Success	Yes
	40	38	KPC-2	–	GEN COL TIG	Success	No
	41	53	KPC-2	Bacteremia	GEN COL FOS	Success	Yes
	42	57	KPC	Bacteremia	GEN COL TIG	Failure	Yes
	43	75	KPC	Bacteremia	FOS COL TIG TZP	Failure	Yes
Peritonitis	44	63	KPC-2	–	AMK TIG CIP	Failure	No
	45	61	KPC-2	–	AMK COL TIG	Success	No
	46	27	KPC-2	Cholangitis	GEN COL TIG	–	Yes
	47	55	KPC-2	–	AMK TZP SMT	Success	No
Surgical site	48	70	KPC-2	Bacteremia	AMK COL TIG		Yes
	49	34	KPC-2	–	ERT FOS COL	Success	No
Ventriculitis	50	43	KPC-2	–	AMK GEN COL TIG	Success	No
Meningitis	51	18	OXA-48	–	FOS COL TZP	Success	No
Lower respiratory	52	39	OXA-48	–	AMK COL TIG	–	No
Prosthetic joint	53	58	KPC	–	AMK COL TIG	Failure	Yes

*bla* beta-lactamase, *SSI* surgical site infection, *UTI* urinary tract infection, *AMK* amikacin, *CIP* ciprofloxacin, *COL* colistin, *DOR* doripenem, *DOX* doxycycline, *ERT* ertapenem, *FOS* fosfomycin, *GEN* gentamicin, *MER* meropenem, *PMB* polymyxin B, *RIF* rifampin, *SMT* sulfamethoxazole/trimethoprim, *TIG* tigecycline, *TZP* piperacillin/tazobactam

#### Carbapenem use

**Fig. 4:**
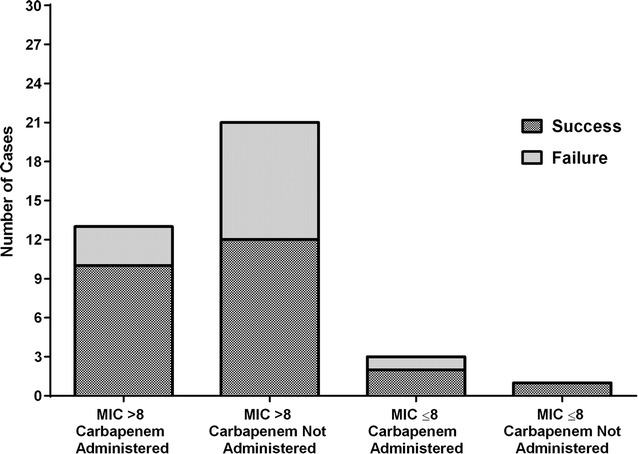
Carbapenem use and success rates stratified by a minimum inhibitory concentration level of 8 mg/L. Failure is a composite of clinical failure, microbiological failure or death

Carbapenem use and mortality stratified by meropenem MIC among tier 1 patients is presented in Fig. 4. The meropenem MIC data were not reported for 15 of the 53 patients. When a carbapenem was administered with a meropenem MIC > 8 mg/L, 10 of 13 (77%) patients were deemed a clinical success. Twenty-one patients were not administered a carbapenem where the meropenem MIC was > 8 mg/mL. Among these patients, 12 of 21 (57%) were deemed a clinical success. In patients where the meropenem MIC was ≤ 8 mg/L, a carbapenem was administered in three cases, which resulted in clinical success in two of the cases.

### Tier 2 results

The remaining ten studies were classified as tier 2 [12, 15, 34–36, 42, 44–46, 48]. Data from these articles were difficult to gather since it was either missing or combined with patients on dual combination or monotherapy. These studies were categorized as tier 2, since triple combination antibiotic therapy was utilized for the treatment of CPKP; however, the patient-specific and clinical details could not be ascertained.

## Discussion

The widespread dissemination of *Klebsiella pneumoniae* carbapenemase producers has resulted in extensive spread of this resistant pathogen across the globe [1, 8]. Our findings show a corresponding increase in the number of publications related to CPKP as resistance began to spread and worsen. This trend in publications was not only limited to Europe and the US, but also included South America and Southeast Asia. Furthermore, these strains are no longer confined to healthcare facilities but have spread to long-term care facilities and even to the community [49–52]. This rapidly growing problem also highlights the urgent need for new therapeutic strategies against resistant isolates. There is a growing interest and need to investigate combination therapies, so the aim of this review was to summarize available clinical data on the role of triple combination therapy in the treatment of infections due to carbapenemase producers.

The most appropriate antibiotic treatment regimens for the treatment of CPKP infections are not well defined. Our results show that triple combination therapy for the treatment of CPKP is being utilized with polymyxin as the common backbone antibiotic. The observed sustained pharmacodynamic in vitro activity of combination antibiotics that prevent the development of resistance has motivated clinicians to explore these promising combinations in their patients [16]. The current clinical treatment of infections due to these strains are largely based on clinical experience and observational studies. The increasing prevalence of carbapenemase-producing Enterobacteriaceae resistant to almost all available agents including polymyxins and tigecycline is highly concerning [53, 54]. This has forced clinicians to reevaluate their treatment strategies against these highly resistant strains that cause infections associated with high mortality rates.

The Consortium on Resistance against Carbapenems in *Klebsiella pneumoniae* (CRACKLE) performed a prospective multicenter study that included 260 patients infected or colonized with carbapenem-resistant *Klebsiella pneumoniae.* The authors found that 39% of patients with bloodstream infection (BSI) or pneumonia died or were discharged to hospice [55]. Additionally, these patients were hospitalized for significantly longer periods, with an increased median total length of hospital stay of 5 and 10 days for BSI and pneumonia, respectively. Tzouvelekis et al. [11] reviewed the efficacy of combination therapy against infections due to carbapenemase-producing Enterobacteriaceae and, based on data from 889 patients, found that combination therapy was effective in 441 (48.6%) patients compared to monotherapy in 346 (38.1%) patients and 102 (11.3%) patients who received inappropriate monotherapy with an antibiotic agent that had no in vitro activity against the infecting pathogen. Furthermore, monotherapy with carbapenem, tigecycline, or colistin were associated with unacceptably high mortality rates of 40.1, 41.1, and 42.8%, respectively, similar to the high mortality of 46.1% observed in patients who received ‘inappropriate’ monotherapy [13]. Our findings are similar to these studies, with triple combination therapy considered clinically successful in 25 of 39 (64%) patients. Also, the overall mortality among patients treated with triple combinations was 35.8%, which is similar to findings from the CRACKLE study. In a study by Qureshi et al. [4], the most commonly used treatment combinations were colistin-polymyxin B or tigecycline combined with a carbapenem. Use of combination therapy resulted in lower mortality of 12.5% (1/8) compared to 66.7% (8/12) with polymyxin, carbapenem, or tigecycline alone [4]. The use of combinations for definitive therapy was associated with improved survival in bacteremia caused by KPC-producing *K. pneumoniae*. We also found that colistin-polymyxin B was the backbone antibiotic to many of these triple combination regimens along with tigecycline, highlighting the importance of polymyxins as a treatment option for these highly resistant organisms.

The increasing emergence of strains resistant to polymyxins and the development of polymyxin resistance on treatment are very concerning [56]. This is especially true as our findings show that polymyxins were commonly used as part of triple combination therapy for treating CPKP. Development of polymyxin resistance with an associated increase in mortality has been reported in multiple studies [14, 15, 57]. The observed association may be due to decreased polymyxin susceptibility or possibly differences in baseline patient characteristics, severity of infection, or lack of adequate empirical therapy. Regardless, the development of polymyxin resistance is alarming. Polymyxin resistance may be mediated by modification of outer membrane lipopolysaccharide or by increased production of capsular polysaccharide in *K. pneumoniae* [58]. Treatment of 12 patients infected with CRKP with polymyxin alone resulted in significant increases in polymyxin MICs for three of the 12 patients in a relatively short period of 5–21 days. This rapid change could be attributed to reinfection with a resistant strain under antibiotic selective pressure. This change in susceptibility was prevented in patients treated with a combination of polymyxin B and tigecycline [59].

A few emerging treatment options for CPKP infections appear promising. The most prominent new agent is ceftazidime–avibactam, a cephalosporin combined with a novel β-lactamase inhibitor approved by the US Food and Drug Administration (FDA) in February 2015 [60]. Ceftazidime–avibactam has shown potent in vitro activity against CRE isolates [61–63]. and there have also been reports that ceftazidime–avibactam is effective for CPKP infections after other combination regimens have failed [19, 64, 65]. Other β-lactam/β-lactamase inhibitor combinations are also being investigated including ceftolozane–tazobactam and aztreonam–avibactam [12, 66]. Plazomicin, a novel aminoglycoside that has shown in vitro activity against CRE, is currently undergoing a Phase 3 clinical trial (NCT01970371) as part of a combination therapy [67]. Another agent showing potential is eravacycline, a tetracycline derivative, which has shown in vitro efficacy against CRE as well as for complicated intra-abdominal infections and complicated urinary tract infections in clinical trials [68, 69].

There are several limitations of this review. First, there were a limited number of cases treated with triple combination therapy that were considered tier 1. The purpose of this review was to explore the utilization of this treatment modality for CPKP infections; therefore we needed to be more stringent in our selection of cases. A number of articles did not provide sufficient detail to explore triple combination therapy and related outcomes, therefore we excluded them from the analysis. Second, the cases are heterogeneous in terms of their resistance mechanisms, MICs and antimicrobial therapies, which may limit the generalizability of our findings. The number of CPKP cases treated with triple antibiotic therapy are limited and it was our intention to include those that provided sufficient detail. Further research is necessary to explore specific antimicrobial regimens based on different levels of resistance for the treatment of CPKP. Finally, the timing of antibiotics differed among the studies with only a small number of patients receiving triple antibiotic therapy as empirical therapy. Accordingly, these differences may influence the clinical outcomes.

## Conclusions

CPKP infections are a significant challenge worldwide, especially as infections caused by these organisms are associated with morbidity and high mortality. Few antimicrobials retain activity against CPKP, so polymyxin-based combinations have become an important treatment option for patients with these infections. Clinical data on the most appropriate antibiotic combination are sparse, and most of the evidence is based on observational studies. This review summarizes how triple combination therapy has been used against carbapenemase-producing *K. pneumoniae* infections. Triple combination therapy is being considered as a treatment option in the clinic and may be appropriate given the rise of polymyxin resistance. Further research is necessary to establish which treatment combination is superior and how to best utilize combination therapy for the treatment of CPKP infections.
